# Effects of HLA-DRB1 alleles on susceptibility and clinical manifestations in Japanese patients with adult onset Still’s disease

**DOI:** 10.1186/s13075-017-1406-x

**Published:** 2017-09-12

**Authors:** Tomoyuki Asano, Hiroshi Furukawa, Shuzo Sato, Makiko Yashiro, Hiroko Kobayashi, Hiroshi Watanabe, Eiji Suzuki, Tomoyuki Ito, Yoshifumi Ubara, Daisuke Kobayashi, Nozomi Iwanaga, Yasumori Izumi, Keita Fujikawa, Satoshi Yamasaki, Tadashi Nakamura, Tomohiro Koga, Toshimasa Shimizu, Masataka Umeda, Fumiaki Nonaka, Michio Yasunami, Yukitaka Ueki, Katsumi Eguchi, Naoyuki Tsuchiya, Shigeto Tohma, Koh-ichiro Yoshiura, Hiromasa Ohira, Atsushi Kawakami, Kiyoshi Migita

**Affiliations:** 1 Department of Rheumatology, Fukushima Medical University School of Medicine, 1 Hikarigaoka, Fukushima, Fukushima 960-1295 Japan; 2 Molecular and Genetic Epidemiology Laboratory, Faculty of Medicine, University of Tsukuba, 1-1-1 Tennodai, Tsukuba, 305-8575 Japan; 3 Department of Rheumatology, Ohta Nishinouchi General Hospital Foundation, 2-5-20 Nishinouchi, Koriyama, Fukushima 963-8558 Japan; 4 Department of Rheumatology Nagaoka Red Cross Hospital, 297-1, Senshu-2, Nagaoka, Niigata 940-2085 Japan; 5 Department of Rheumatology, Toranomon Hospital, Toranomon 2-2-2, Minato-ku, Tokyo 105-8470 Japan; 6 Department of Rheumatology, Niigata Rheumatic Center, 1-2-8 Honcho, Shibata, Niigata 957-0054 Japan; 7 Clinical Research Center, NHO Nagasaki Medical Center, Kubara 2-1001-1, Omura, Nagasaki 856-8562 Japan; 8 Department of Rheumatology, Japan Community Health care Organization, Isahaya General Hospital, Eishohigashi-machi 24-1, Isahaya, 854-8501 Japan; 9 Department of Rheumatology, Kurume University Medical Center, Kokubu 155-1 1-2-3, Kurume, 734-8551 Japan; 10 Department of Rheumatology, Sakurajyuji Hospital, Miyukibe 1-1-1, Kumamoto, 861-4173 Japan; 11 Department of Immunology and Rheumatology, Unit of Translational Medicine, Graduate School of Biomedical Sciences, Nagasaki University, Sakamoto1-7-1, Nagasaki, 852-8501 Japan; 12 Departments of Rheumatology, Sasebo City General Hospital, Hirase 9-3, Sasebo, 857-8511 Japan; 13 Department of Medical Genomics, Life Science Institute, Saga-ken Medical Centre Koseikan, 400 Kasemachi-Nakabaru, Saga, 840-8571 Japan; 14 Department of Rheumatology, Sasebo Chuo Hospital, Yamato 15, Sasebo, 857-1195 Japan; 15 Clinical Research Center for Allergy and Rheumatology, National Hospital Organization Sagamihara National Hospital, 18-1 Sakuradai, Minami-ku, Sagamihara 252-0392 Japan; 16 Department of Human Genetics, Atomic Bomb Disease Institute, Nagasaki University, Sakamoto 1-12-4, Nagasaki, 852-8523 Japan; 17 Department of Gastroenterology, Fukushima Medical University School of Medicine, 1 Hikarigaoka, Fukushima, Fukushima, 960-1295 Japan

**Keywords:** Adult onset Still’s disease, Autoinflammatory disease, Human leukocyte antigen, Macrophage-activation syndrome, Systemic juvenile idiopathic arthritis

## Abstract

**Background:**

*HLA-DRB1* alleles are major determinants of genetic predisposition to rheumatic diseases. We assessed whether *DRB1* alleles are associated with susceptibility to particular clinical features of adult onset Still’s disease (AOSD) in a Japanese population by determining the *DRB1* allele distributions.

**Methods:**

*DRB1* genotyping of 96 patients with AOSD and 1,026 healthy controls was performed. Genomic DNA samples from the AOSD patients were also genotyped for *MEFV* exons 1, 2, 3, and 10 by direct sequencing.

**Results:**

In Japanese patients with AOSD, we observed a predisposing association of DRB1*15:01 (*p* = 8.60 × 10^−6^, corrected *p* (*P*c) = 0.0002, odds ratio (OR) = 3.04, 95% confidence interval (95% CI) = 1.91–4.84) and DR5 serological group (*p* = 0.0006, OR = 2.39, 95% CI = 1.49–3.83) and a protective association of DRB1*09:01 (*p* = 0.0004, *P*c = 0.0110, OR = 0.34, 95% CI = 0.18–0.66) with AOSD, and amino acid residues 86 and 98 of the DRβ chain were protectively associated with AOSD. *MEFV* variants were identified in 49 patients with AOSD (56.3%). The predisposing effect of DR5 was confirmed only in patients with AOSD who had *MEFV* variants and not in those without *MEFV* variants. Additionally, DR5 in patients with AOSD are associated with macrophage activation syndrome (MAS) and steroid pulse therapy.

**Conclusion:**

The DRB1*15:01 and DR5 are both associated with AOSD susceptibility in Japanese subjects. A protective association between the DRB1*09:01 allele and AOSD was also observed in these patients. Our data also highlight the effects of *DRB1* alleles in susceptibility to AOSD.

**Electronic supplementary material:**

The online version of this article (doi:10.1186/s13075-017-1406-x) contains supplementary material, which is available to authorized users.

## Background

Adult onset Still’s disease (AOSD), manifested by spiking fever, skin rash, and arthritis, is a complex (multigenic) autoinflammatory disease, meaning that its development depends on a combination of genetic and environmental risk factors [1]. The concept of autoinflammation originally emerged from innate immune system activation [2]. Although there are rare monogenic forms of autoinflammatory disease, acquired autoinflammatory diseases in which both innate and adaptive immune abnormalities are implicated are more common [3]; this group includes genetically complex rheumatic diseases, such as AOSD [4]. Complex genetic factors may confer susceptibility to AOSD development under certain environmental conditions [5].

The *HLA* cluster is one of the most intensively studied genetic factors in AOSD [6]. HLA molecules are linked with adaptive immunity, where they act to present peptide antigens to antigen receptors on T lymphocytes [7]. Currently, there is no consensus on the relationship between AOSD and *HLA*. Previous studies on the genetic predisposition to AOSD have limitations and have had inconsistent results [8]. This may be caused by the small number of patients included in these studies, due to the rarity of this disease and its heterogeneous clinical manifestations.

Systemic juvenile idiopathic arthritis (sJIA) and AOSD both manifest as systemic arthritis [9]. Recent studies have provided new clues to the genetic underpinning of sJIA. A genome-wide association study of sJIA identified a strong association between sJIA and HLA-DRB1*11 [10]. This study supports a role for *DRB1* alleles as a major sJIA risk factor [10]. To determine whether or not genetic variations of *DRB1* alleles also influence AOSD risk, we investigated the *DRB1* alleles in Japanese patients with AOSD. Previously, we found preliminary evidence suggesting that familial Mediterranean fever (FMF)-related *MEFV* variants can affect the disease phenotype of AOSD [11]. In this study we evaluated the influence of the *MEFV* genotype and HLA-DRB1 alleles on AOSD risk.

## Methods

### Design, setting, patients, and measurements

We retrospectively evaluated 96 patients (mean age ± standard deviation = 50.2 ± 20.2 years, 78 female (81.2%)), who had AOSD and were treated at the Department of Rheumatology of the participating hospital group, between 2010 and 2017. The patients had all been diagnosed with AOSD according to the Yamaguchi criteria [12], after exclusion of infectious, hematologic, and autoimmune diseases. Among 96 patients, 87 patients had undergone laboratory tests, including a complete blood count, liver function tests, urinalysis, erythrocyte sedimentation rate (ESR), C-reactive protein (CRP) and ferritin. In the remaining nine patients, the demographic and *MEFV* genotyping data were not available except gender and age. The clinical characteristics of these patients, including age, gender, length of follow up, clinical features, treatments, outcomes, and complications, were evaluated using a standardized form. The study considered three clinical AOSD courses: (1) monocyclic, defined as a single episode that subsequently faded and was followed by persistent good health for 1 year or more of follow up; (2) polycyclic, defined as a complete remission followed by one or more exacerbations; and (3) chronic, defined as persistently active disease, usually associated with polyarthritis [13]. Complicated AOSD was defined as including one or more of the following conditions: liver dysfunction, disseminated intravascular coagulation, and macrophage activation syndrome (MAS) as defined previously [14]. This study was approved by the institutional review board of the Sasebo City Hospital (number 2012-A-22) and participating hospitals.

### Genotyping methods

*DRB1* allele typing was performed using a Luminex 200 system (Luminex, Austin, TX, USA) and a WAKFlow HLA Typing kit (Wakunaga, Hiroshima, Japan) as described previously [15]. *DRB1* alleles were assigned automatically using WAKflow Typing software (Wakunaga). The DR5 serological group consists of DRB1*11:01, *12:01, and *12:02 [16]*.* Genotyping results for the healthy controls (mean age ± standard deviation = 37.7 ± 11.7 years, 303 male (29.8%)) were previously reported [17].

### *MEFV* gene analysis

Genomic DNA was extracted from whole blood using the Promega Wizard® Genomic DNA Purification Kit (Madison, WI, USA) according to the manufacturer’s instructions. Polymerase chain reaction (PCR) was performed using forward and reverse primers for each exon of the *MEFV* gene, as described previously [18]. PCR was performed using the forward and reverse primers for each exon (Additional file 1: Figure S1). The resulting PCR products were purified using ExoSAP-IT (GE Healthcare, Tokyo, Japan) and directly sequenced using specific primers and BigDye Terminator v1.1 (Applied Biosystems, Tokyo, Japan). The *MEFV* genetic analysis was approved by the Ethics Committee of Fukushima Medical University School of Medicine (2016, number 2920).

### Statistical analysis

Differences among AOSD characteristics were analyzed by performing the Mann-Whitney *U* test or chi-squared test using 2 × 2 contingency tables. The association of allele carrier frequencies, haplotype carrier frequencies or amino acid residue carrier frequencies with AOSD was analyzed using Fisher’s exact test with 2 × 2 contingency tables under the dominant model. Adjustment for multiple comparisons was conducted with the Bonferroni method; corrected *p* (*P*c) values were calculated by multiplying the *p* value by the number of tested alleles or amino acid positions. To examine whether each *DRB1* allele independently contributes to AOSD, multiple logistic regression analysis under the additive model was employed and the deviation from 0 was evaluated for coefficients using the Wald test.

## Results

### Demographic findings of enrolled patients with AOSD

Clinical manifestations of the enrolled patients are shown in Table 1. Among the 87 enrolled patients with AOSD, 69 (79.3%) were women, and the mean age at diagnosis was 49.3 ± 19.9 years. In terms of the disease course, 56 patients (64.3%) had the polycyclic systemic type, 19 (21.8%) had the monocyclic systemic type, and 12 (13.7%) had the chronic articular type of AOSD (Table 1).

**Table 1:** Demographics of AOSD patients

Variable	Value
Number	87
Female, *n* (%)	69 (79.3)
Age at onset (years)	49.3 ± 19.9
Ferritin (ng/mL)	11251.2 ± 16997.9
CRP (mg/dL)	12.4 ± 8.2
ESR (mm(1 h))	71.1 ± 30.1
Liver dysfunction, *n* (%)	68 (78.1)
MAS, *n* (%)	23 (26.4)
Initial dose of PSL (mg/day)	41.8 ± 12.2
Steroid pulse, *n* (%)	55 (63.2)
Immunosuppressant, *n* (%)	51 (58.6)
Biologics, *n* (%)	26 (29.8)
Polycyclic systemic type, *n* (%)	56 (64.3)
Monocyclic systemic type, *n* (%)	19 (21.8)
Chronic arthritis type, *n* (%)	12 (13.7)
Relapse, *n* (%)	40 (45.9)

Mean ± standard deviation or number (percentage) is shown

*AOSD* adult onset Still’s disease, *CRP* C-reactive protein, *ESR* erythrocyte sedimentation rate, *MAS* macrophage activation syndrome, *PSL* prednisolone

### Association of specific *DRB1* alleles with AOSD

Associations of specific *DRB1* alleles with AOSD were evaluated in a case-control study consisting of the 96 Japanese patients with AOSD as described previously and 1026 healthy subjects (Table 2). The patients with AOSD had significantly higher frequencies of the DRB1*15:01 allele (*p* = 8.60 × 10^−6^, corrected *p* (*P*c) = 0.0002, odds ratio (OR) = 3.04, 95% confidence interval (95% CI) = 1.91–4.84) compared with healthy subjects. Although patients with AOSD appeared to have higher frequencies of DRB1*12:01 allele than healthy subjects, this difference was not statistically significant. However, an association between DR5 serological group and AOSD was observed.

**Table 2:** *HLA-DRB1* allele carrier frequency in the patients with AOSD and in controls

	Cases (n = 96)	Controls (n = 1026)	*P*	OR	*P*c	95% CI
DRB1*01:01	9 (9.4)	110 (10.7)	0.8622	0.86	NS	(0.42–1.76)
DRB1*03:01	0 (0.0)	3 (0.3)	1.0000	1.52	NS	(0.08–29.55)
DRB1*04:01	0 (0.0)	22 (2.1)	0.2486	0.23	NS	(0.01–3.84)
DRB1*04:03	4 (4.2)	47 (4.6)	1.0000	0.91	NS	(0.32–2.57)
DRB1*04:04	0 (0.0)	4 (0.4)	1.0000	1.18	NS	(0.06–22.03)
DRB1*04:05	21 (21.9)	243 (23.7)	0.8014	0.90	NS	(0.54–1.49)
DRB1*04:06	2 (2.1)	76 (7.4)	0.0562	0.27	NS	(0.06–1.10)
DRB1*04:07	0 (0.0)	15 (1.5)	0.6313	0.34	NS	(0.02–5.70)
DRB1*04:10	4 (4.2)	32 (3.1)	0.5413	1.35	NS	(0.47–3.90)
DRB1*07:01	0 (0.0)	9 (0.9)	1.0000	0.55	NS	(0.03–9.61)
DRB1*08:02	13 (13.5)	72 (7.0)	0.0403	2.08	NS	(1.10–3.90)
DRB1*08:03	16 (16.7)	153 (14.9)	0.6544	1.14	NS	(0.65–2.01)
DRB1*08:09	0 (0.0)	2 (0.2)	1.0000	2.12	NS	(0.10–44.55)
DRB1*09:01	11 (11.5)	280 (27.3)	0.0004	0.34	0.0110	(0.18–0.66)
DRB1*10:01	0 (0.0)	5 (0.5)	1.0000	0.96	NS	(0.05–17.54)
DRB1*11:01	6 (6.3)	41 (4.0)	0.2837	1.60	NS	(0.66–3.87)
DRB1*12:01	16 (16.7)	75 (7.3)	0.0031	2.54	0.0896	(1.41–4.56)
DRB1*12:02	8 (8.3)	37 (3.6)	0.0488	2.43	NS	(1.10–5.38)
DRB1*13:01	2 (2.1)	8 (0.8)	0.2082	2.71	NS	(0.57–12.93)
DRB1*13:02	5 (5.2)	163 (15.9)	0.0040	0.29	0.1150	(0.12–0.73)
DRB1*14:03	7 (7.3)	44 (4.3)	0.1941	1.76	NS	(0.77–4.01)
DRB1*14:04	0 (0.0)	4 (0.4)	1.0000	1.18	NS	(0.06–22.03)
DRB1*14:05	6 (6.3)	40 (3.9)	0.2758	1.64	NS	(0.68–3.98)
DRB1*14:06	2 (2.1)	29 (2.8)	1.0000	0.73	NS	(0.17–3.11)
DRB1*14:07	0 (0.0)	2 (0.2)	1.0000	2.12	NS	(0.10–44.55)
DRB1*14:54	6 (6.3)	58 (5.7)	0.8168	1.11	NS	(0.47–2.65)
DRB1*15:01	31 (32.3)	139 (13.5)	8.60 × 10^-6^	3.04	0.0002	(1.91–4.84)
DRB1*15:02	18 (18.8)	224 (21.8)	0.5197	0.83	NS	(0.48–1.41)
DRB1*16:02	0 (0.0)	18 (1.8)	0.3919	0.28	NS	(0.02–4.72)
DR5(*11, *12)	28 (29.2)	151 (14.7)	0.0006	2.39		(1.49–3.83)

Allele carrier frequencies are shown in parentheses (%). Association was tested by Fisher’s exact test using 2 × 2 contingency tables under the dominant model. *AOSD* adult onset Still’s disease, *OR* odds ratio, *CI* confidence interval, *P*c corrected *p* value, *NS* not significant

### Reduced DRB1*09:01 allele carrier frequencies in AOSD

In contrast to the predisposing effects of the DRB1*15:01 allele, DRB1*09:01 was negatively associated with AOSD (Table 2). In fact, although the DRB1*15:01 allele predisposed individuals to AOSD in the absence of DRB1*09:01, the DRB1*15:01 allele was not associated with AOSD in those carrying the DRB1*09:01 allele (Table 3). Thus, the protective effects of DRB1*09:01 can neutralize the AOSD-predisposing effects of the DRB1*15:01 allele. When the allele carrier frequencies of *DRB1* alleles in patients with AOSD were compared with age-matched healthy controls, a similar associative tendency was observed (Additional file 2: Table S1). In addition, conditional logistic regression analysis between age, gender, DRB1*09:01, DRB1*15:01, and DR5 in AOSD was performed (Additional file 3: Table S2). The association of DRB1*09:01, DRB1*15:01, and DR5 remained significant, when conditioned on age or gender, and these were independent of each other.

**Table 3:** *HLA-DRB1* genotype frequencies in the patients with AOSD and in controls

	Cases (n = 96)	Controls (n = 1026)	*P*	OR	95% CI
*15:01/any alleles	31 (32.3)	139 (13.5)	8.60 × 10^-6^	3.04	(1.91-4.84)
*15:01/*09:01	1 (1.0)	21 (2.0)	1.0000	0.50	
*15:01/alleles other than *09:01	30 (31.3)	118 (11.5)	1.64 × 10^-6^	3.50	(2.18-5.61)

Genotype frequencies are shown in parentheses (%). Association was tested by Fisher’s exact test using 2 × 2 contingency tables. *AOSD* adult onset Still’s disease, *OR* odds ratio, *CI* confidence interval

### *MEFV* variant analysis

All patients with AOSD were successfully genotyped for the *MEFV* gene. *MEFV* variants were identified in 49 patients with AOSD (56.3%), and the distributions of *MEFV* variants in the patients are shown in Table 4. We stratified the patients with AOSD according to the presence of *MEFV* variants. The predisposing effect of DRB1*15:01 on AOSD development was not changed by the presence or absence of *MEFV* variants (Table 5). In contrast, the AOSD-predisposing effect of DR5 was confirmed in patients with AOSD with *MEFV* variants, but DR5 was not associated with AOSD in patients without *MEFV* variants. Thus, the presence or absence of *MEFV* variants can affect the AOSD-predisposing effects of DR5.

**Table 4:** *MEFV* genotypes of patients with AOSD

Mutations	Number of patients (percentage) (n = 87)
M694I/normal	2 (2.3)
G632S/E408Q	1 (1.1)
P369S/R408Q	4 (4.6)
E148Q/P369S	1 (1.1)
E148Q/E148Q/P369S/R408Q	1 (1.1)
L110P/E148Q/E148Q/P369S/R408Q	1 (1.1)
E148Q/P369S/R408Q	2 (2.3)
E148Q/R202Q	1 (1.1)
R202Q/normal	3 (3.4)
E148Q/normal	19 (21.8)
E148Q/E148Q	2 (2.3)
L110P/E148Q	6 (6.9)
L110P/E148Q//E148Q	2 (2.3)
L110P/E148Q//R202Q	1 (1.1)
L110P/L110P/E148Q//E148Q	1 (1.1)
E84K/normal	1 (1.1)
E84K/L110P/E148Q	1 (1.1)
Normal	38 (43.7)

*AOSD* adult onset Still’s disease

**Table 5:** *HLA-DRB1* allele carrier frequency in the patients with AOSD with or without *MEFV* variants

	AOSD with MEFV variants	AOSD without MEFV variants	Controls	AOSD with MEFV variants vs. controls				AOSD without MEFV variants vs. controls			
	(n = 49)	(n = 38)	(n = 1026)	*P*	OR	*P*c	95% CI	*P*	OR	*P*c	95% CI
DRB1*15:01	17 (34.7)	13 (34.2)	139 (13.5)	0.0002	3.39	0.0068	(1.83–6.27)	0.0013	3.32	0.0388	(1.66–6.64)
DR5(DRB1*11, *12)	16 (32.7)	8 (21.1)	151 (14.7)	0.0019	2.81		(1.51–5.23)	0.2543	1.55		(0.70–3.43)

Allele carrier frequencies are shown in parentheses (%). Association was tested by Fisher’s exact test using 2 × 2 contingency tables under the dominant model. *AOSD* adult onset Still’s disease, *OR* odds ratio, *CI* confidence interval, *Pc* corrected *p* value, *NS* not significant

### Association of specific *DRB1* alleles and clinical manifestations of AOSD

To evaluate the potential correlation between *DRB1* alleles and the clinical manifestations of AOSD, we stratified the patients with AOSD according to the presence of the DRB1*15:01 allele (Table 6). There was no significant difference in the clinical manifestations, including the disease phenotype, between patients with AOSD with and without the DRB1*15:01 allele. We then compared the clinical manifestations between patients with AOSD with or without DR5. Patients with AOSD with DR5 more frequently have macrophage activation syndrome (MAS) and are more frequently treated by steroid pulse therapy than those without DR5 (Table 6).

**Table 6:** Comparison of the demographics between patients with AOSD with or without DRB1*15:01 or *DR5*

	DRB1*15:01 (+)	DRB1*15:01 (-)	*P*	*DR5* (+)	*DR5* (-)	*P*
Number	29	58		23	64	
Female, *n* (%)	24 (82.7)	45 (77.5)	0.7789	20 (86.9)	49 (77.4)	0.2912
Age at onset (years)	46.6 ± 20.3	50.6 ± 19.6	0.3980	50.6 ± 20.5	48.1 ± 19.7	0.7469
Ferritin (ng/mL)	12818.5 ± 18292.9	10555.5 ± 16284.7	0.3657	12669.4 ± 16305.9	10802.9 ± 17224.9	0.3243
CRP (mg/dL)	11.9 ± 8.2	12.7 ± 8.3	0.6802	12.0 ± 9.2	12.6 ± 7.9	0.5068
ESR (mm(1 h))	59.6 ± 25.3	75.4 ± 30.9	0.1289	67.4 ± 23.0	72.5 ± 32.5	0.9361
Liver dysfunction, *n* (%)	24 (82.7)	44 (75.8)	0.4630	19 (82.6)	49 (76.5)	0.5472
MAS, *n* (%)	10 (38.6)	13 (22.4)	0.2288	10 (43.4)	13 (20.3)	0.0307
Initial dose of PSL (mg/day)	43.8 ± 13.0	41.5 ± 11.7	0.4379	46.1 ± 10.9	40.3 ± 12.4	0.1184
Steroid pulse, *n* (%)	17 (58.6)	38 (65.5)	0.4987	19 (82.6)	36 (56.2)	0.0245
Immunosuppressant, *n* (%)	18 (57.6)	33 (56.8)	0.6442	15 (65.2)	36 (56.2)	0.4539
Biologics, *n* (%)	10 (38.6)	16 (27.5)	0.5077	6 (26.0)	20 (31.2)	0.6427
Polycyclic systemic type, *n* (%)	20 (68.9)	36 (62.0)	0.5266	18 (78.2)	38 (59.3)	0.1048
Monocyclic systemic type, *n* (%)	8 (27.5)	13 (22.4)	0.5951	4 (17.3)	17 (26.5)	0.3780
Chronic arthritis type, *n* (%)	4 (13.7)	8 (13.7)	1.0000	2 (8.6)	10 (15.6)	0.4085
Relapse, *n* (%)	14 (48.2)	26 (44.8)	0.7619	11 (47.8)	29 (45.3)	0.8357

Association was tested between patients with adult onset Still’s disease (AOSD) with or without DRB1*15:01 or DR5 analyzed by the chi-squared test using 2 × 2 contingency tables or the Mann-Whitney *U* test. *CRP* C-reactive protein, *ESR* erythrocyte sedimentation rate, *MAS* macrophage activation syndrome, *PSL* prednisolone

### Amino acid residues in the HLA-DRβ chain are protectively associated with AOSD

**Fig. 1:**
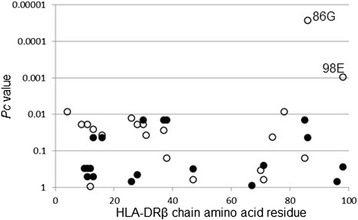
Association between amino acid residues in the DRβ chain and adult onset Still’s disease. Differences in amino acid residue carrier frequencies were analyzed by Fisher’s exact test using 2 × 2 contingency tables. Corrected *p* (*P*c) values were calculated by multiplying the *p* value by the number of amino acid residues tested. Predisposing association is indicated by filled circles and protective association by open circles

We also analyzed the association between amino acid residues in the HLA-DRβ chain and AOSD. Glycine at position 86 (86G, *p* = 7.88 × 10^-7^, OR = 0.24, *P*c = 2.68 × 10^-5^, 95% CI 0.15–0.41) and glutamic acid at position 98 (98E, *p* = 2.80 × 10^-5^, OR = 0.40, *P*c = 0.0010, 95% CI 0.26–0.62) in the DRβ chain showed a protective association with AOSD (Fig. 1, open circle).

## Discussion

AOSD is an inflammatory disorder of uncertain etiology characterized by spiking fever, rash, and arthritis that is accompanied by high ferritin levels [19]. Both sJIA and AOSD are considered to be autoinflammatory diseases with a dysregulated innate immune system [20]. However, this characterization is limited by the fact that dysfunctions of the innate and adaptive immune systems are not mutually exclusive, even in monogenic forms of autoinflammatory disease [21].

Certain *HLA* alleles have been reported to confer a predisposition to rheumatic diseases [22]. Although no hereditary trend has been reported in AOSD, some studies have reported the associations between HLA-DR and AOSD. Pouchot et al. described a strong association between AOSD and HLA-DR2 [23]. Wouters et al. found an association between DRB1*04 and AOSD and reported that DR6 (DRB1*13, *14) was more frequent in cases with axial joint involvement [24]. Miller et al. reported a frequent association between AOSD and HLA-DR7 [6]. Fujii et al. evaluated the major histocompatibility complex (MHC) class II alleles in 30 Japanese patients with AOSD and reported that DRB1*15:01 and DRB1*12:01 were more frequently detected in subjects with chronic articular forms of AOSD than in healthy controls [25]. Additionally, a Korean study showed that patients with AOSD more frequently had DRB1*12 and *15, and less frequently had DRB1*04 than control subjects [26]. However, controversies remain on the association between HLA-DR molecules and AOSD.

Recent studies have provided new clues to the genetic predispositions for developing systemic arthritis. A genome-wide association study of sJIA patients identified a strong association between sJIA and DRB1*11 [10]. Interestingly, Ombrello et al. raised the new idea that the MHC may act not as an antigen-presenting molecule but rather as a direct activator of MHC class-expressing macrophages or dendritic cells [10]. Our data show that both DRB1*15:01 and DR5 are associated with AOSD in Japanese patients. DRB1*15:01 is the HLA allele that was most strongly associated with AOSD in our study, and these results are consistent with those of previous studies in Japanese or Korean patients [25, 26]. Conversely, the DRB1*09:01 allele had a significantly deceased frequency in patients with AOSD when compared with that in control subjects as reported in multiple sclerosis [27, 28]. However, this negative association of AOSD with the DRB1*09:01 allele needs to be confirmed in the other ethnic populations because the distribution of HLA alleles is different between Japanese and other ethnic populations. Notably, extensive heterogeneity is observed in the clinical manifestations of AOSD, suggesting that AOSD may actually be a heterogeneous subset of disorders [29]. Our results suggest that *DRB1* alleles may play a role in the occurrence of AOSD; however, it is possible that there are transcriptional changes for other *HLA* loci that are co-inherited with *DRB1*, and these haplotypes may also contribute to the AOSD risk.

It was found that amino acid residues 86 and 98 of the DRβ chain were protectively associated with AOSD (Fig. 1). The DRB1*09:01 allele includes these two amino acid residues, suggesting the predominant protective effects of DRB1*09:01 on AOSD. Amino acid residues 85, 86, 89, and 90 form the HLA-DR peptide-binding groove for peptide position 1 [30], suggesting the involvement of peptide antigens bound to the specific HLA-DR molecule to prevent AOSD.

*MEFV* variants are commonly found in the Japanese population [31]. Although the significance of *MEFV* variants is controversial, some studies have suggested that they can influence the phenotypes of inflammatory disorders [32]. Here, we found that the DR5-associated risk for AOSD was not detected in Japanese Patients with AOSD without *MEFV* variants, whereas the DR5-associated risk was preserved in Japanese patients with *MEFV* gene variants. These finding suggest a possible interaction between *DRB1* and *MEFV* in AOSD development or in the AOSD disease phenotype. Furthermore, these findings imply that differences between susceptible and non-susceptible DR5 alleles are complemented by *MEFV* variants in Japanese patients with AOSD, which could be explained by the broad involvement of pyrin, the *MEFV* encoded protein, in the regulation of the inflammasome and inflammatory processes.

Rheumatic diseases are likely on the spectrum between autoimmunity and autoinflammation [33]. It was suggested that a pathogenic model in which autoinflammatory processes beget an autoimmune condition, sJIA, is a prototype of AOSD [34]. A genome-wide association study on sJIA identified a strong association between sJIA and DRB1*11 alleles. DR5 is a broad-antigen serotype that is further split into HLA-DR11, which includes DRB1*11:01, and HLA-DR12 [17]. In our study, patients with DR5 were more frequently associated with AOSD with macrophage activation syndrome (MAS), which has some similarities to the clinical features of sJIA. These findings are consistent with the reports by Ombrello et al. that demonstrate an association between sJIA and DRB1*11 alleles [11]. From a pathogenic point of view, most autoinflammatory and autoimmune diseases share a chronic aberrant activation of the immune system, which leads to tissue inflammation and/or damage [35].]. In contrast to autoimmune diseases, “autoinflammatory diseases” present with inflammation in the absence of either high-titer autoantibodies or autoreactive T cells. Although the evidence for autoimmunity in AOSD is scant, there are some data that support a role for T cells in its pathogenesis [36]. Additionally, class II HLA molecules have a role in innate immunity and in the regulation of macrophages, including antigen-presenting cells. For example, super-antigen-engaged antigen-presenting cells induce proinflammatory cytokines [37].

There are limitations in our study. Control subjects were younger than the patients. AOSD is a rare disorder offsetting any potential bias due to development of AOSD later in life by a younger control. Another limitation is that all of our study subjects were Japanese because too few patients from other racial/ethnic backgrounds were available for analysis. Also, the number of patients with AOSD was modest and a larger study would be needed.

## Conclusion

Our study provides evidence that DRB1*15:01 and DR5 confer a risk for AOSD. A protective association between the DRB1*09:01 allele and AOSD was also observed in this study. Specifically, our data demonstrate that DR5 is associated with AOSD complicated with MAS, in a Japanese population. Additional studies are needed to determine the specific mechanism through which the HLA-DRB1 influences the risk of AOSD.

## Additional files

Additional file 1: Figure S1. Primers and polymerase chain reaction conditions. (JPG 171 kb)

Additional file 2: Table S1. HLA-DRB1 allele carrier frequency in the patients with AOSD and age-matched healthy controls (1:2). (PDF 57 kb)

Additional file 3: Table S2. Conditional logistic regression analysis between the protective HLA alleles in AOSD. (PDF 43 kb)

## Abbreviations

AOSD: adult onset Still’s disease
HLA: human leukocyte antigen
MAS: macrophage-activation syndrome
MHC: Major histocompatibility class
*P*c: Corrected *p* value
sJIA: systemic juvenile idiopathic arthritis
