# Supplementary figures and images for: Effects of HLA-DRB1 alleles on susceptibility and clinical manifestations in Japanese patients with adult onset Still’s disease
Source: Arthritis Res Ther. 2017 Sep 12;19:199. doi: 10.1186/s13075-017-1406-x (PMC5596459; doi:10.1186/s13075-017-1406-x)

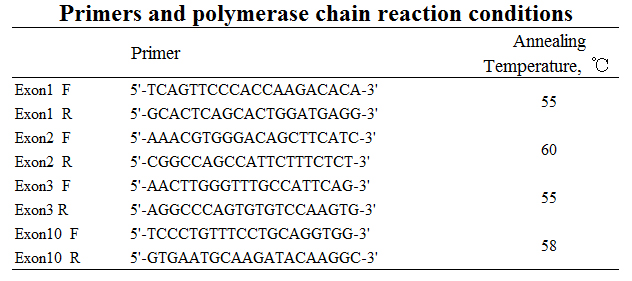

Supplement: Supplementary file 1 — Primers and polymerase chain reaction conditions. (JPG 171 kb) [file 13075_2017_1406_MOESM1_ESM.jpg]
