# Supplementary material for: Effects of HLA-DRB1 alleles on susceptibility and clinical manifestations in Japanese patients with adult onset Still’s disease
Source: Arthritis Res Ther. 2017 Sep 12;19:199. doi: 10.1186/s13075-017-1406-x (PMC5596459; doi:10.1186/s13075-017-1406-x)
Supplement: Supplementary file 2 — HLA-DRB1 allele carrier frequency in the patients with AOSD and age-matched healthy controls (1:2). (PDF 57 kb) [file 13075_2017_1406_MOESM2_ESM.pdf]

Table S1. *HLA-DRB1* allele carrier frequency in the AOSD patients and age-matched healthy controls (1:2).

	Case (n=69)	Control (n=138)	*P*	OR	*P*c	95%CI
*DRB1**01:01*	6 (8.7)	19 (13.8)	0.3686	0.60	NS	(0.23-1.57)
*DRB1**04:01*	0 (0.0)	4 (2.9)	0.3036	0.22	NS	(0.01-4.05)
*DRB1**04:03*	4 (5.8)	4 (2.9)	0.4450	2.06	NS	(0.50-8.51)
*DRB1**04:05*	12 (17.4)	38 (27.5)	0.1231	0.55	NS	(0.27-1.15)
*DRB1**04:06*	2 (2.9)	12 (8.7)	0.1486	0.31	NS	(0.07-1.44)
*DRB1**04:07*	0 (0.0)	2 (1.4)	0.5534	0.39	NS	(0.02-8.30)
*DRB1**04:10*	2 (2.9)	6 (4.3)	0.7215	0.66	NS	(0.13-3.34)
*DRB1**07:01*	0 (0.0)	3 (2.2)	0.5523	0.28	NS	(0.01-5.47)
*DRB1**08:02*	8 (11.6)	8 (5.8)	0.1697	2.13	NS	(0.76-5.95)
*DRB1**08:03*	13 (18.8)	20 (14.5)	0.4268	1.37	NS	(0.64-2.95)
*DRB1**09:01*	7 (10.1)	31 (22.5)	0.0360	0.39	0.8291	(0.16-0.94)
*DRB1**11:01*	4 (5.8)	5 (3.6)	0.4850	1.64	NS	(0.43-6.30)
*DRB1**12:01*	13 (18.8)	8 (5.8)	0.0060	3.77	0.1388	(1.48-9.61)
*DRB1**12:02*	6 (8.7)	4 (2.9)	0.0870	3.19	NS	(0.87-11.71)
*DRB1**13:02*	5 (7.2)	19 (13.8)	0.2488	0.49	NS	(0.17-1.37)
*DRB1**14:03*	5 (7.2)	5 (3.6)	0.3063	2.08	NS	(0.58-7.44)
*DRB1**14:05*	4 (5.8)	6 (4.3)	0.7340	1.35	NS	(0.37-4.97)
*DRB1**14:06*	1 (1.4)	5 (3.6)	0.6659	0.39	NS	(0.04-3.42)
*DRB1**14:07*	0 (0.0)	1 (0.7)	1.0000	0.66	NS	(0.03-16.40)
*DRB1**14:54*	5 (7.2)	11 (8.0)	1.0000	0.90	NS	(0.30-2.71)
*DRB1**15:01*	26 (37.7)	18 (13.0)	0.0001	4.03	0.0025	(2.01-8.07)
*DRB1**15:02*	11 (15.9)	37 (26.8)	0.1151	0.52	NS	(0.25-1.09)
*DRB1**16:02*	0 (0.0)	1 (0.7)	1.0000	0.66	NS	(0.03-16.40)
DR5(*11, *12)	21 (30.4)	17 (12.3)	0.0022	3.11		(1.51-6.41)

Allele carrier frequencies are shown in parenthesis (%). Association was tested by Fisher's exact test using 2x2 contingency tables under the dominant model. AOSD: adult onset still disease, OR: odds ratio, CI: confidence interval, Pc: corrected P value, NS: not significant. Mean age ± SD of AOSD patients and healthy controls were 46.8 ± 15.9 and 46.2 ± 15.4 years, respectively.
