# Supplementary material for: Effects of HLA-DRB1 alleles on susceptibility and clinical manifestations in Japanese patients with adult onset Still’s disease
Source: Arthritis Res Ther. 2017 Sep 12;19:199. doi: 10.1186/s13075-017-1406-x (PMC5596459; doi:10.1186/s13075-017-1406-x)
Supplement: Supplementary file 3 — Conditional logistic regression analysis between the protective HLA alleles in AOSD. (PDF 43 kb) [file 13075_2017_1406_MOESM3_ESM.pdf]

Table S2. Conditional logistic regression analysis between the protective HLA alleles in AOSD

HLA allele	Unconditioned		Conditioned on gender		Conditioned on age		Conditioned on *DRB1*09:01*		Conditioned on *DRB1*15:01*		Conditioned on DR5	
	P	OR (95%CI)	$P_{adjusted}$	$OR_{adjusted}$ (95%CI)	$P_{adjusted}$	$OR_{adjusted}$ (95%CI)	$P_{adjusted}$	$OR_{adjusted}$ (95%CI)	$P_{adjusted}$	$OR_{adjusted}$ (95%CI)	$P_{adjusted}$	$OR_{adjusted}$ (95%CI)
*DRB1*09:01*	0.0010	0.35 (0.19−0.65)	0.0013	0.36 (0.20−0.67)	0.0020	0.32 (0.16−0.66)	NA	NA	0.0031	0.39 (0.21−0.73)	0.0030	0.39 (0.21−0.73)
*DRB1*15:01*	0.0001	2.35 (1.55−3.55)	0.0001	2.36 (1.56−3.58)	$2.17X10^{-5}$	2.88 (1.77−4.68)	0.0004	2.11 (1.39−3.20)	NA	NA	$1.35X10^{-5}$	2.55 (1.67−3.88)
DR5	$2.28X10^{-5}$	2.39 (1.60−3.58)	$2.31X10^{-5}$	2.41 (1.60−3.61)	$2.20X10^{-5}$	2.93 (1.78−4.82)	0.0002	2.17 (1.44−3.26)	$6.07X10^{-6}$	2.58 (1.71−3.90)	NA	NA

AOSD: adult onset still’s disease, OR: odds ratio, CI: confidence interval, NA not applicable. P, OR, 95%CI, Padjusted , ORadjusted were calculated by logistic regression analysis under the additive model.
